# Arthroscopic Subscapularis Augmentation for the Treatment of Bankart Lesions: A Narrative Review of Indications, Techniques, and Comparative Outcomes

**DOI:** 10.3390/jcm15207921

**Published:** 2026-10-14

**Authors:** Jaewook Park, Jaesung Yoo

**Affiliations:** Department of Orthopedic Surgery, Dankook University Hospital, 201 Manghyang-ro, Dongnam-gu, Cheonan 31116, Republic of Korea; osshoulder@gmail.com

**Keywords:** anterior shoulder instability, Bankart repair, subscapularis tenodesis, glenoid bone loss, arthroscopic subscapularis augmentation, Latarjet procedure, recurrence, arthroscopy

## Abstract

Recurrent anterior shoulder instability from a Bankart lesion is conventionally treated with arthroscopic Bankart repair, but recurrence remains substantial in patients with glenoid bone loss (GBL), capsular deficiency, hyperlaxity, or contact-sport participation. Arthroscopic subscapularis augmentation (ASA) tenodeses the upper third of the subscapularis to the anterior glenoid alongside Bankart repair, providing an additional anterior soft-tissue restraint. This narrative review, based on a literature search updated to September 2026, summarizes the rationale, indications, techniques, postoperative management, and outcomes of ASA. Selected observational cohorts, mostly from overlapping author groups, have reported recurrence of approximately 2.7–7.0% in high-risk patients with subcritical GBL; because these figures are compared mainly with historical series, the incremental benefit of ASA over appropriately selected comparator procedures remains uncertain. In one multicenter comparison, ASA had single-digit recurrence and fewer complications than open Latarjet, whereas Latarjet procedures had the lowest recurrence. ASA combined with bone grafting is an evolving option within the range of GBL studied so far (approximately 15–30%), not an established Latarjet equivalent. The evidence is predominantly retrospective Level III–IV with short- to mid-term follow-up, and prospective comparative studies with standardized outcome definitions are needed.

## 1. Introduction

The Bankart lesion—detachment of the anteroinferior glenoid labrum and capsuloligamentous complex—is considered the essential lesion underlying recurrent anterior shoulder instability, and arthroscopic Bankart repair remains the standard surgical treatment. However, the reliability of isolated Bankart repair depends strongly on concomitant bone loss. In early series, recurrence after arthroscopic repair alone reached 67% in patients with substantial glenoid or humeral bone deficiency [1], and patients scoring above the high-risk threshold of the Instability Severity Index had a recurrence risk of approximately 70% [2,3]. These classic figures come from cohorts enriched for large bony defects and therefore represent a worst-case contrast. More contemporary estimates in comparable bone-loss bands are lower but remain clinically important: a systematic review of 528 patients with glenoid bone loss treated with isolated arthroscopic Bankart repair reported a pooled recurrence rate of 23.7% and identified glenoid bone loss of 16% or more as predictive of failure [4], and even “subcritical” bone loss above approximately 13.5% has been associated with inferior patient-reported outcomes [5]. The interaction between glenoid and humeral-sided (Hill-Sachs) bone loss is further captured by the on-track/off-track classification, which helps predict engagement and guides the choice between soft-tissue and bony stabilization procedures [6]. Younger age at surgery is an established risk factor and is included in the Instability Severity Index [2,3], and risk factors for recurrence have also been examined specifically in patients younger than 30 years [7]. Where an off-track or engaging Hill-Sachs lesion is present without substantial glenoid bone loss, remplissage—filling the humeral head defect with the infraspinatus tendon and posterior capsule—is a widely used adjunct [8], although anchor fixation in this technique can be compromised when the humeral bone quality is poor, such as in acute or acute-on-chronic fracture-associated dislocations.

For more severe glenoid bone loss, the Latarjet coracoid transfer procedure is frequently regarded as the reference standard, with systematic reviews and meta-analyses reporting pooled recurrence rates of approximately 11.6% for Latarjet versus 21.1% for Bankart repair [9], and long-term comparative data confirming significantly lower redislocation rates after open Latarjet than after arthroscopic Bankart repair, at the cost of a higher complication rate [10]. The mechanical basis of the Latarjet procedure’s effectiveness is understood to derive substantially from a dynamic “sling effect” of the conjoint tendon across the lower subscapularis as the arm approaches the position of apprehension [11], a concept that traces back to Symeonides’ cadaveric observation that elongation or dysfunction of the subscapularis muscle contributes to recurrent anterior dislocation [12]. Building on this biomechanical principle, arthroscopic subscapularis augmentation (ASA) was introduced as a soft-tissue technique that reinforces the Bankart repair through tenodesis of the upper third of the subscapularis tendon to the anterior glenoid, thereby providing an additional anterior soft-tissue restraint with a proposed sling-like effect, without transferring bone.

## 2. Methods

This narrative review summarizes the conceptual rationale, indications, technical variants, postoperative management, and outcomes of ASA for the treatment of Bankart lesions. PubMed/MEDLINE was searched from inception to 28 September 2026, supplemented by Google Scholar and manual screening of the reference lists of retrieved articles; the initial search covered publications through December 2024 and was repeated with the same search strategy before resubmission. Key terms were combined with Boolean operators as follows: (“subscapularis augmentation” OR “subscapularis tenodesis”) AND (“Bankart” OR “shoulder instability” OR “shoulder dislocation”); (“dynamic anterior stabilization” OR “long head of the biceps”) AND “shoulder instability”; and (“bone graft” OR “bone block” OR “allograft” OR “xenograft”) AND “subscapularis”. Peer-reviewed English-language clinical series, comparative studies, case reports, technique articles, and biomechanical studies describing ASA or closely related sling procedures were eligible; conference abstracts, non-English articles, and reports without extractable outcome data were excluded. Titles and abstracts were screened by one author (J.P.), the full texts of potentially relevant articles were assessed by both authors, and disagreements were resolved by discussion. Data were extracted by one author (J.P.) and checked against the original publications by the second author (J.Y.). In total, 14 clinical series, 1 case report, 5 technique articles, and 2 biomechanical studies were included; additional references were used for background. Studies of biceps-based dynamic anterior stabilization were retained for mechanistic and technical context and were not combined with ASA outcome data. Generative artificial intelligence (Claude Opus 4.6; Anthropic, San Francisco, CA, USA) was used to assist with literature searching and summarization; all AI-assisted output was independently checked by the authors against the original source publications.

We did not perform a meta-analysis because outcome instruments (Rowe, ASES, WOSI, Constant, UCLA) and reporting formats (means, medians, or proportions of “excellent” results) differ across studies, recurrence was defined inconsistently, and glenoid-bone-loss thresholds and measurement methods vary. These factors argue against quantitative pooling, but they would not by themselves preclude a systematic search. We chose a narrative selection and synthesis because the aim of the review was to map the rationale, technical evolution, and indications of ASA, which required technique and biomechanical articles that outcome-based systematic eligibility criteria would not usually capture. The findings should therefore be read as a structured narrative overview rather than a systematic review. The design and main characteristics of each clinical series are summarized in Section 11 to allow readers to judge their quality and independence.

## 3. Concept and Biomechanical Rationale: The Subscapularis Sling Effect

ASA was first described by Maiotti and Massoni as an arthroscopic technique for capsulolabral deficiency, in which the upper one-third of the subscapularis tendon is perforated several millimeters from its superior border and tenodesed to the anterior glenoid neck alongside the standard Bankart repair [13]. Conceptually, the technique is described as producing a triple effect: re-tensioning of an elongated or attenuated upper subscapularis, direct augmentation of a deficient capsulolabral complex, and restoration of coracohumeral ligament tension, without shortening the subscapularis tendon as a whole and therefore without markedly restricting external rotation.

A cadaveric biomechanical study demonstrated that ASA was more effective than isolated Bankart repair at resisting anteroinferior humeral head translation [14], supporting the mechanical plausibility of the sling concept in a controlled laboratory setting. A related but mechanistically distinct dynamic-sling strategy, Dynamic Anterior Stabilization using the long head of the biceps tendon, has also been tested biomechanically: in a controlled cadaveric study across simulated 10% and 20% anterior glenoid defects, this technique produced significantly less anterior humeral head translation than isolated Bankart repair under increasing posterior loads [15], suggesting that a dynamic musculotendinous sling can partially compensate for glenoid bone deficiency that would otherwise necessitate bony reconstruction.

**Figure 1:**
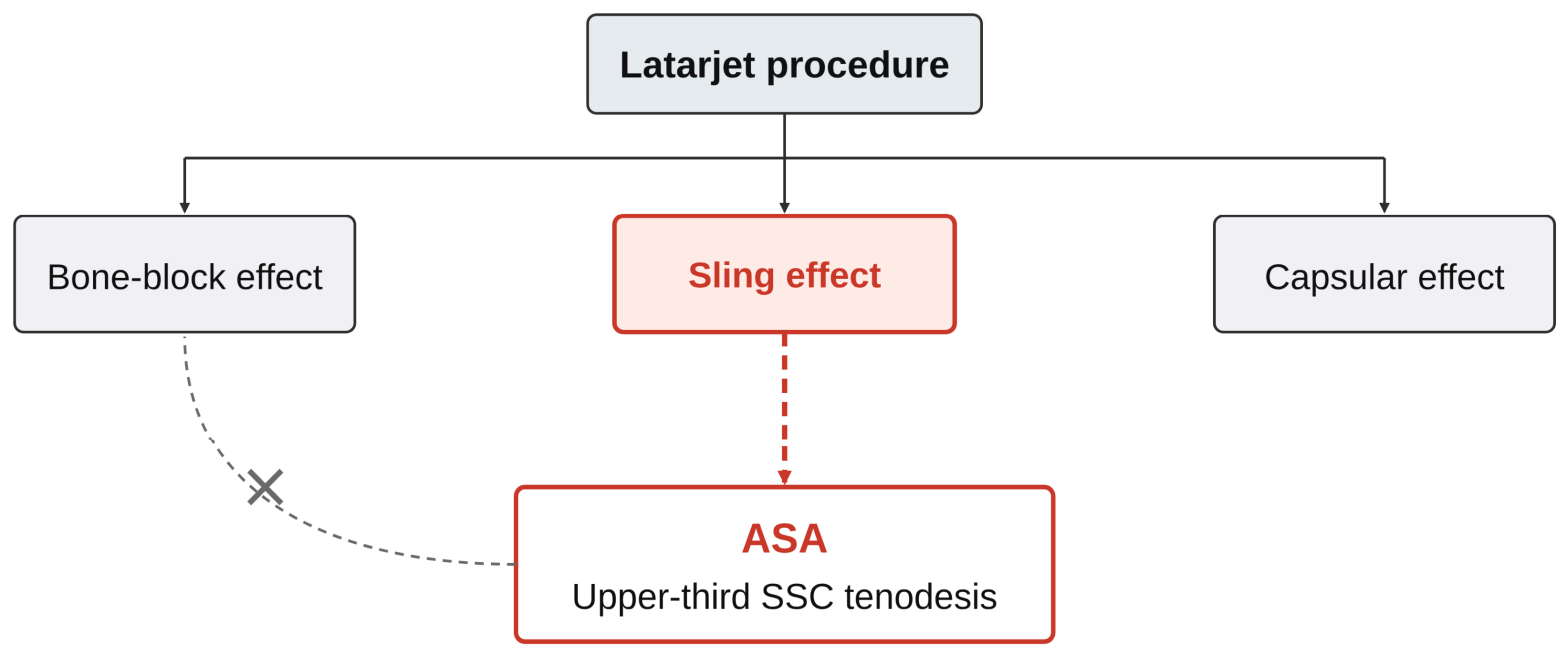
Proposed conceptual relationship between arthroscopic subscapularis augmentation (ASA) and the stabilizing mechanisms of the Latarjet procedure. Of the three proposed Latarjet mechanisms (bone-block, sling, and capsular effects), the sling effect of the conjoint tendon was identified as a major contributor in cadaveric testing [11]. ASA tenodeses the upper third of the subscapularis to the anterior glenoid and is proposed to provide a sling-like soft-tissue restraint (dashed arrow); this is a conceptual relationship rather than an established mechanical equivalence, and ASA does not reproduce the bone-block effect (crossed dashed line). SSC, subscapularis.

These laboratory findings support biomechanical plausibility but have important limitations. In the ASA study, eight cadaveric shoulders with a 15–20% anterior glenoid defect were tested under a 30 N translational load at 0°, 30°, and 60° of glenohumeral abduction, with the scapula fixed and without simulated muscle forces; ASA reduced anterior translation at 30° and 60° of abduction but also reduced external rotation by approximately 44° and 49° at 0° and 30° of abduction, and the results reflect the immediate postoperative state [14]. Because the subscapularis was not actively loaded, a dynamic sling was not tested. Moreover, ASA and the Latarjet procedure are anatomically different constructs: ASA tenodeses the upper subscapularis to the anterior glenoid, whereas the Latarjet sling involves the transferred conjoint tendon acting across the lower subscapularis [11]. ASA is therefore best described as providing an additional anterior soft-tissue restraint with a proposed sling-like effect, conceptually related to—but not established as mechanically equivalent to—the conjoint-tendon mechanism of the Latarjet procedure; it may also act through the re-tensioning and capsulolabral effects described above. This proposed relationship is summarized in Figure 1.

## 4. Indications for Subscapularis Augmentation

**Figure 2:**
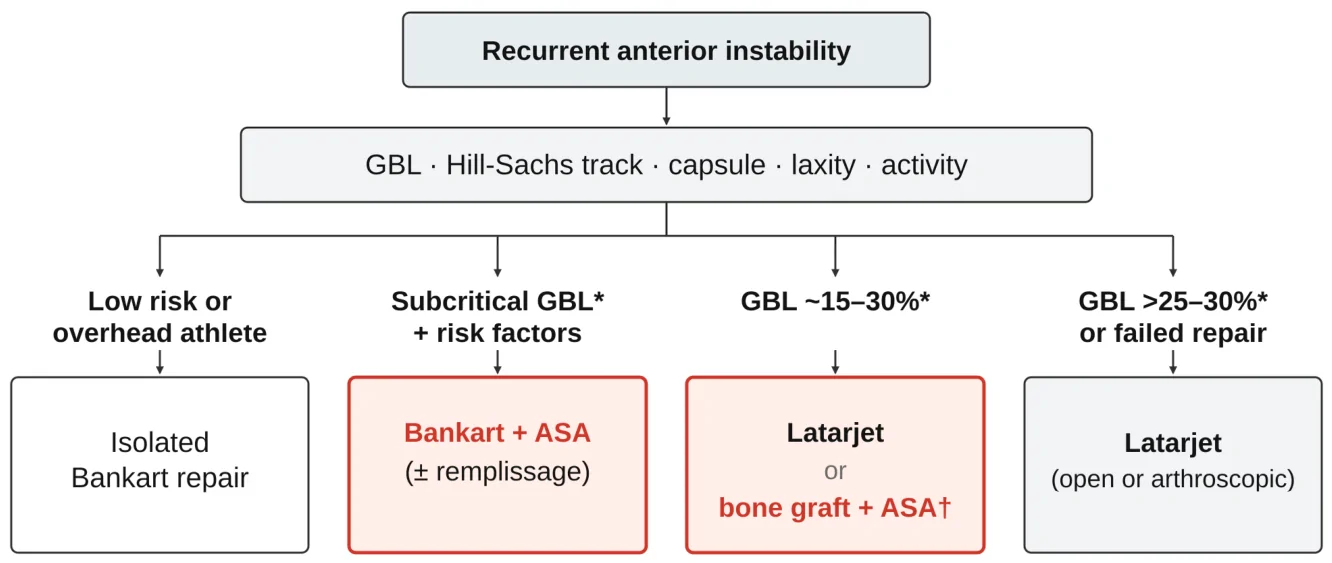
Illustrative treatment framework synthesized from the reviewed literature, organized by glenoid bone loss (GBL) and patient-specific risk factors (Table 1). Risk factors include capsular deficiency, generalized hyperlaxity, and contact or collision sports; multidirectional or voluntary instability and glenohumeral osteoarthritis are generally excluded. * GBL bands overlap because eligibility cut-offs differ between studies (e.g., <13.5%, <15%, <20%, <25%, and 15–30%) and were obtained with non-standardized measurement methods. † Evolving option supported by small series; not an established Latarjet equivalent. This framework is a narrative synthesis, not a validated clinical algorithm or consensus recommendation, and the inclusion of a procedure does not imply demonstrated superiority over alternatives.

Across the published literature, the indication for ASA consistently centers on the combination of (a) capsulolabral or capsular tissue deficiency, often after failed prior repair or in the setting of chronic instability; (b) subcritical-to-moderate glenoid bone loss; (c) frequently an accompanying, non-critical Hill-Sachs lesion; and (d) participation in contact or collision sports, or documented generalized hyperlaxity. Throwing and overhead athletes are consistently excluded across series because of concern that even modest external-rotation restriction could impair the cocking phase of overhead motion, and multidirectional instability, voluntary instability, and pre-existing glenohumeral osteoarthritis are uniformly listed as exclusion criteria (Table 1). The glenoid-bone-loss thresholds listed in Table 1 (from under 13.5% to under 25% for soft-tissue ASA, and 15–30% for bone-graft-combined procedures) are study-specific eligibility criteria, not validated or consensus treatment thresholds. Moreover, the method used to quantify bone loss is not standardized across the cited series: the Maiotti–Russo studies mostly used the Pico surface-area method on computed tomography (CT) [16,17,18,19,20], whereas other series used different or incompletely described methods, and Pico, Sugaya, best-fit-circle, two- and three-dimensional CT, and magnetic resonance imaging (MRI) measurements are not interchangeable. For orientation only, these indications are organized into an illustrative framework in Figure 2; this figure collapses heterogeneous study-specific cut-offs and is a narrative synthesis, not a validated clinical algorithm.

## 5. Surgical Technique Variants

Since its original description, several technical variants of ASA have been reported. To avoid transferring evidence from one construct to another, Table 2 groups them into four categories: (A) native-tendon ASA, in which the upper subscapularis itself is tenodesed and the variants differ in fixation (suture anchor, combined tendon–capsule passage, or transglenoid cortical button); (B) graft-modified ASA, which interposes a tendon graft; (C) bone-graft-combined ASA, in which the tenodesis is combined with a glenoid bone block for more severe bone loss; and (D) biceps-based dynamic anterior stabilization, which reroutes the long head of the biceps tendon (LHBT) through the subscapularis. Although group D shares the sling concept, it is a different operation, and its outcome data are not combined with ASA data in this review.

**Figure 3:**
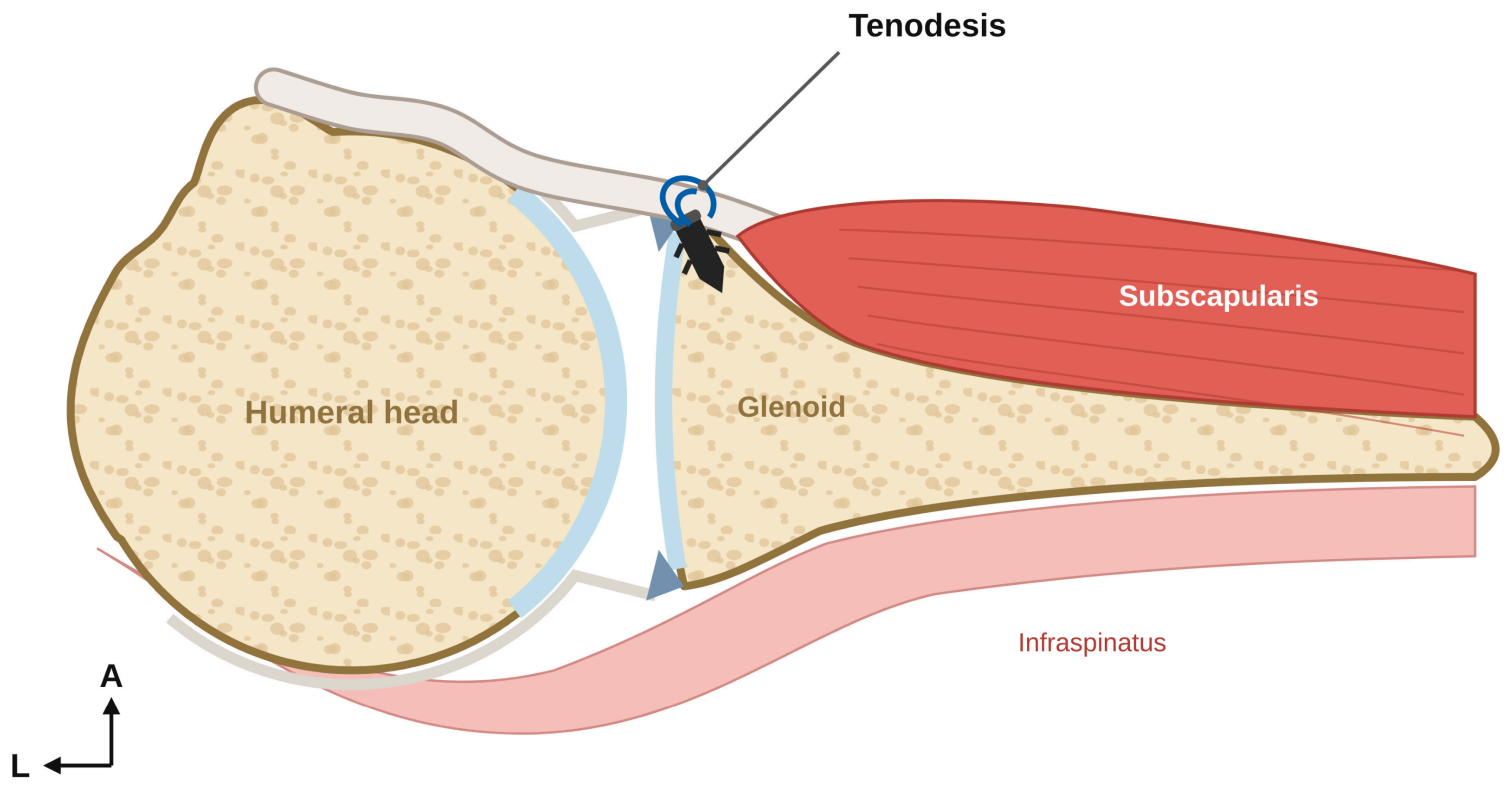
Schematic axial view of arthroscopic subscapularis augmentation (right shoulder). The subscapularis tendon is tenodesed to the anterior glenoid margin with a single suture anchor, creating a dynamic anterior restraint that resists anterior translation of the humeral head in abduction and external rotation. Technical variants are summarized in Table 2. The black symbol indicates the suture anchor, and the blue loop indicates the suture securing the tenodesis. Drawing is schematic and not to scale. A, anterior; L, lateral.

In the original technique, after standard Bankart repair with suture anchors along the anteroinferior glenoid rim, the shoulder is positioned in external rotation and the upper one-third of the subscapularis tendon is captured together with the labrum using additional suture anchors placed more superiorly on the glenoid neck, effectively extending the repair construct cephalad and creating a dynamic checkrein as the arm moves toward abduction and external rotation (Figure 3). Bone-graft-combined variants supplement this soft-tissue step with an arthroscopically placed iliac crest autograft, allograft, or xenograft bone block, secured with cortical buttons or a posterior cerclage guide, thereby addressing both the bony and soft-tissue deficits in patients with more substantial glenoid bone loss. Fixation variants replace the tenodesis anchor with a transglenoid tunnel and an adjustable-loop cortical button [28] or pass the subscapularis tendon and anteroinferior capsule together as a single composite layer [22]. Biceps-based dynamic anterior stabilization, by contrast, uses the tenotomized LHBT rather than the native subscapularis as the sling tissue [15,31].

## 6. Postoperative Rehabilitation and Expected Motion Loss

Because ASA is a tenodesis of the upper subscapularis to the glenoid neck, postoperative management aims to protect both the tenodesis and the Bankart repair while limiting permanent loss of external rotation. The published protocols are broadly similar and derive mainly from the originating centers. In the Maiotti series, the arm was immobilized in a sling or brace in adduction for 4 weeks; passive and active range-of-motion exercises, including gradual external rotation, began at approximately week 5; full range of motion was targeted between weeks 6 and 8; strengthening and proprioceptive training started at weeks 8–9; and sport-specific training began at approximately week 10 [16,17,18]. Return to contact and collision sports was permitted at 4 months in the earlier series [16,17] and at 4–6 months in the hyperlaxity cohort [18]. Independent series used a similar 4-week immobilization period [25,32], whereas return to sport was deferred to 5 months in the ASA arm of the Latarjet comparison [19] and to 6 months after xenograft bone-block augmentation [20].

Some loss of external rotation should be anticipated and discussed with patients preoperatively. Relative to the contralateral shoulder, mean external-rotation deficits ranged from 6° to 15° with the arm at the side and from 3° to 8° in 90° of abduction across the Maiotti–Russo series [16,17,18,19,20]; in each series, the deficit was greater with the arm at the side than in abduction. An independent single-surgeon series reported a mean external-rotation loss of 10.2° [22], adding ASA to Bankart repair with remplissage produced a significantly greater external-rotation deficit than remplissage alone [33], and a comparative study found no significant difference in postoperative range of motion between modified ASA and Bankart repair alone [29]. These deficits are generally considered acceptable for contact and collision athletes, but they underlie the consistent exclusion of throwing and overhead athletes. Standardized, prospectively reported rehabilitation milestones and return-to-play criteria are not yet available for ASA, and the optimal timing of external-rotation exercises after tenodesis remains undefined.

## 7. Comparative Recurrence Rates: ASA Versus Standard Bankart Repair

The central clinical rationale for ASA is a reduction in recurrence relative to isolated Bankart repair in comparable high-risk patients (bone loss, hyperlaxity, contact-sport participation). An important caveat is that this comparison is mostly indirect: ASA series are usually contrasted with historical or pooled estimates for isolated repair—up to 67% in classic high-risk series and approximately 24% in a contemporary pooled analysis of patients with glenoid bone loss [1,2,3,4]—rather than with concurrent controls (Table 3). Across the principal ASA case series, recurrence has ranged from approximately 2.7% to 7.0%, generally accompanied by substantial improvement in Rowe and other functional outcome scores. However, the Maiotti series published in 2016, 2017, 2021, and 2023 share surgeons and centers, and the first three had overlapping enrolment periods between 2009 and 2017 [16,17,18,21]; the original reports do not state whether any patient was included in more than one publication, so overlap can be neither confirmed nor excluded, and the 2.7–7.0% range should not be read as a set of independent samples. An independent series from outside the Maiotti–Russo network supports a similar recurrence band: among 34 patients with glenoid bone loss below 13.5% treated with Bankart repair plus ASA, 2 had a redislocation at a mean follow-up of 42 months [26]. Two recent comparative studies from other independent groups reported similar short-term results. In 56 patients with GBL <15%, recurrence was 0% after Bankart repair with remplissage plus ASA (*n* = 17) and 5.1% after Bankart repair with remplissage alone (*n* = 39), at the cost of a significantly greater external-rotation deficit [33]; and in 50 patients with GBL of 13.5–20%, no recurrent dislocation occurred at 1 year after either Bankart repair plus ASA or LHBT transposition plus subscapularis augmentation [34]. Only two small studies provide concurrent comparisons with isolated Bankart repair alone [25,29]. Outcome definitions also differ: most series counted only redislocation, whereas subluxation, subjective instability, persistent apprehension, revision surgery, and composite failure were reported inconsistently or not at all, so recurrence rates are not directly interchangeable across studies (Table 3).

In a retrospective comparison specifically designed to isolate the added value of subscapularis augmentation, modified ASA combined with capsulolabral reconstruction produced numerically superior outcomes to Bankart repair alone in patients with glenoid bone loss under 25%, without a significant difference in postoperative range of motion between groups [29]. Similarly, in a single-surgeon case series of 83 patients with capsulolabral (ligament-labral) failure and glenoid bone loss under 25% (mean 21%), a technique variant in which the subscapularis tendon and anteroinferior capsule were passed together as a single composite layer produced significant improvements in Constant-Murley (39.7 → 81.2), ASES (39.9 → 81.3), Rowe (33.3 → 82.6), and UCLA (13.2 → 27) scores (all *p* = 0.001), with a recurrence rate of 2.4% (2/83) and a mean external rotation loss of 10.2° [22]. By contrast, a comparative study restricted to non-athletes with moderate glenoid bone loss under 20% found no recurrence in either the subscapularis-augmented or the isolated capsulolabral repair group (0/15 in each) at a mean 48-month follow-up [25]; with 15 patients per group and no events, this observation is imprecise and cannot be taken as a reliable estimate of the underlying recurrence risk. Nevertheless, the subscapularis-augmented group in that study also showed higher objective Rowe scores (median 100 vs. 75, *p* < 0.001) and lower (better) WOSI scores (211.0 vs. 379.9, *p* = 0.018), without a significant difference in ASES scores. This single small comparative study (15 patients per arm) should be interpreted with caution: the SB group’s median Rowe score sat exactly at the scale ceiling (100), raising the possibility of a ceiling effect rather than a robust demonstration of clinical superiority, and no comparable finding has been replicated elsewhere. This isolated observation should not, on its own, be taken as a basis for extending the ASA indication to low-risk, non-athlete patients; it is noted here descriptively rather than as supporting evidence for a broader recommendation. Overall, selected observational cohorts have reported low recurrence after ASA combined with Bankart repair in patients otherwise considered at elevated risk of failure with soft-tissue repair alone—contact/collision athletes, patients with hyperlaxity, and those with capsular deficiency—but the incremental benefit over appropriately selected comparator procedures remains uncertain and should be tested in prospective controlled comparisons.

## 8. Bone-Graft-Combined Subscapularis Augmentation Versus the Latarjet Procedure

For glenoid bone loss beyond the range typically addressed by soft-tissue ASA alone, combining subscapularis augmentation with glenoid bone grafting has been proposed as an alternative to the Latarjet procedure. Direct comparisons between ASA and the Latarjet procedure are limited to two retrospective studies from the originating group. In a comparative series of 20 patients treated with ASA and 20 patients treated with open Latarjet for moderate glenoid bone loss (5–23%), no statistically significant difference was found between groups in Rowe or Constant scores at approximately 20 months; no redislocation occurred after ASA (one transient subluxation), whereas one recurrence after Latarjet was related to coracoid nonunion [19] (Table 4). With 20 patients per group, the absence of a statistically significant difference does not establish equivalence or non-inferiority. A larger multicenter study of 226 patients with GBL <15% and a high Instability Severity Index score (>3) compared four techniques (Bankart plus ASA, Bankart plus remplissage, open Latarjet, and arthroscopic Latarjet): recurrence was 7.0% (4/57) with ASA, 6.1% (4/65) with remplissage, 1.5% (1/66) with open Latarjet, and 0% (0/38) with arthroscopic Latarjet, while excellent Rowe outcomes were achieved in 89.2%, 79.9%, 98.5%, and 81.6% of patients, respectively, and open Latarjet carried the highest complication burden (approximately 15%) [21]. Baseline WOSI scores differed substantially between groups (838 in the ASA group vs. 1456 in the open Latarjet group), the reported comparisons are unadjusted for these differences, and follow-up ranged from 2 to 6 years; these data therefore establish neither equivalence nor a definitive difference between procedures. In this comparison, Latarjet-family procedures had the lowest recurrence, and open Latarjet had the highest rate of excellent Rowe outcomes. In this specific subcritical bone-loss and risk-severity range, the soft-tissue techniques (ASA and remplissage) had single-digit recurrence with fewer complications than open Latarjet; they may therefore be reasonable options for selected patients, but the comparative evidence is insufficient to recommend them as first-line procedures before a bony procedure or as equivalent substitutes for the Latarjet procedure.

For critical bone loss (mean 20%, range 15–30%), combining an arthroscopic iliac crest bone allograft with subscapularis upper-third tenodesis produced excellent Rowe outcomes in 17 of 19 patients (89%) at a mean 34.6-month follow-up, with significant improvement in Constant (82.9 → 88.9, *p* = 0.002), Rowe (25.3 → 89.1, *p* < 0.001), UCLA (23.7 → 31.5, *p* < 0.001), and VAS (3.2 → 1.3, *p* < 0.001) scores, and a recurrence rate of 11% (2/19) [23]. However, bone graft resorption was observed radiographically in all 19 patients (complete in 10, partial in 9). The authors found no statistically significant association between resorption and redislocation and attributed the maintained stability primarily to scar-tissue consolidation of the anterior capsule and the sling effect of the subscapularis tenodesis [23]. With only 19 patients and 2 recurrences, however, that analysis was underpowered to exclude such an association. Universal graft resorption should therefore be regarded primarily as a limitation of the bony component of this technique: stability after complete resorption, if confirmed, is compatible with the sling/scar hypothesis but does not show that the graft is unnecessary. A xenograft-based variant, in which a glenoid bone block is secured with a posterior cerclage guide and combined with ASA, reported no recurrent dislocation and no reduction in graft volume on CT at 24 months in 20 patients with a mean glenoid bone loss of 18.5% [20,24]. These bone-graft-combined ASA techniques should be regarded as an evolving option within the range of glenoid bone loss studied so far (approximately 15–30%), not as an established Latarjet equivalent, given the small sample sizes, short follow-up, universal allograft resorption in at least one series, and the predominance of single-institution or closely affiliated research groups reporting these outcomes.

## 9. Complication Profile: Bone-Graft-Combined Subscapularis Augmentation Versus Latarjet

Any comparison of complication rates between ASA and the Latarjet procedure is made on unequal footing. The favorable complication data for ASA, with or without bone grafting, derive almost entirely from small, single- or dual-institution series performed by the technique’s originating authors, whereas the Latarjet complication literature is drawn from much larger, more heterogeneous, multi-decade pooled cohorts that inevitably capture a wider range of surgeon experience. With this caveat, the comparative complication burden remains a key consideration when weighing bone-graft-combined ASA against the Latarjet procedure. In the largest systematic review of Bristow-Latarjet outcomes (45 studies, 1904 shoulders, 90% open technique), the pooled overall complication rate was 30%, with a 7% unplanned reoperation rate; hardware-related complications specifically have been reported in 6.5% of open cases and up to 17.6% of arthroscopic cases [35]. Recognized Latarjet-specific complications include neurovascular injury (axillary, musculocutaneous, or suprascapular nerve), coracoid graft nonunion or fracture, hardware-related pain or impingement, secondary subscapularis weakness from splitting or rerouting the conjoint tendon, and progressive glenohumeral osteoarthritis from graft malpositioning. By contrast, in the arthroscopic iliac crest allograft plus subscapularis tenodesis series, the authors reported no perioperative complications requiring conversion to open surgery, no graft fracture, no clinically significant graft malpositioning, and no neurovascular events, attributing this favorable safety profile to the graft being introduced through the rotator interval rather than requiring coracoid osteotomy or a subscapularis split [23]. In the four-procedure multicenter comparison, the open Latarjet arm had the highest complication rate of the four techniques studied (approximately 15%), numerically exceeding the rates reported for the ASA and remplissage soft-tissue augmentation arms [21] (Table 5).

Complication reporting standards also differ: graft resorption, although observed in 100% of patients in the allograft series, was not classified by the authors as a complication, whereas analogous graft-related radiographic findings after Latarjet (osteolysis, nonunion) are typically counted as such in the Latarjet literature. Furthermore, contemporary high-volume Latarjet centers with dedicated neuromonitoring and standardized technique have reported markedly lower complication rates (e.g., an overall 90-day complication rate of 4.3% in a 441-patient series, chiefly minor hematomas, with no neurologic or hardware complications) [36], indicating that much of the historical complication burden attributed to Latarjet may be mitigated by surgeon experience and modern technique refinement rather than being an immutable feature of the procedure itself. Bone-graft-combined ASA nonetheless offers a biologically plausible mechanism for a lower complication ceiling—avoidance of coracoid transfer, subscapularis splitting, and large-diameter metallic screw fixation, relying instead on low-profile cortical buttons and suture-based constructs—that merits confirmation in adequately powered, multicenter, surgeon-matched comparative studies before it can be considered a definitively safer alternative rather than a promising but preliminary observation.

## 10. Possible Future Applications: Seizure-Related Instability

A possible future application is in patients with seizure disorders, in whom recurrent dislocation and poor humeral or glenoid bone quality frequently coexist and in whom remplissage may be technically limited by the risk of anchor pull-out in fractured or osteopenic bone. The present authors reported a single case in which a 21-year-old man sustained a seizure-related anterior shoulder dislocation with a large, off-track Hill-Sachs lesion and an acute fracture pattern with poor bone quality at the humeral defect; because of concern about both external-rotation restriction and anchor pull-out with remplissage, ASA combined with Bankart repair was performed instead, with no recurrence of the operated shoulder over more than two years of follow-up, including through a subsequent seizure episode, whereas the untreated contralateral shoulder subsequently dislocated [27]. Together with the broader literature documenting an elevated recurrence risk after shoulder stabilization surgery in patients with epilepsy [37,38,39,40], this observation is hypothesis-generating only: ASA might serve as an anchor-sparing alternative in this population, but no comparative data exist, and seizure-related instability should not be regarded as an established indication for ASA.

## 11. Limitations of the Current Evidence and Future Directions

Several limitations temper the interpretation of the ASA literature. First, essentially all clinical outcome data are Level III–IV retrospective case series or retrospective comparative studies; no randomized controlled trial has directly compared ASA with isolated Bankart repair, remplissage, or the Latarjet procedure. Second, the great majority of clinical series originate from a small number of overlapping author groups (predominantly Maiotti, Russo, and their collaborators) with shared surgeons, centers, and enrolment periods, which raises the possibility of duplicate patients and of publication and selection bias and limits generalizability across surgeons and healthcare settings. Table 6 summarizes the design, recruitment period, centers, comparator, follow-up, and recurrence definition of each clinical series. Because the original reports do not state whether patients were included in more than one publication, overlap among the Maiotti cohorts can be neither confirmed nor excluded, and these reports should not be treated as independent replications. Independent reports are fewer and smaller, including series from China, Turkey, and Greece [22,26,29,33,34], a small non-athlete comparison [25], and a 2025 series of 36 patients from Bangladesh that illustrates the geographic diffusion of the technique but provides only short-term follow-up and limited methodological detail [32]. Several key studies also disclose consulting relationships with the manufacturer (Arthrex) of the anchors used in the described techniques [16,17,18,20]; although disclosed, these relationships warrant consideration when interpreting the uniformly favorable outcomes reported. Third, follow-up durations in most series are short to intermediate (approximately 20–45 months), so long-term recurrence, the durability of the sling effect, and the incidence of secondary glenohumeral osteoarthritis remain incompletely characterized. Fourth, glenoid-bone-loss thresholds (from under 13.5% to under 25%) and measurement methods are not uniform across studies, and recurrence definitions differ (dislocation only versus inclusion of subluxation or apprehension), which complicates direct cross-study comparison and precludes meaningful pooling of recurrence data. Finally, evidence supporting the use of ASA in seizure-related instability currently rests on a single case report, together with indirect epidemiologic data on recurrence after stabilization surgery in patients with epilepsy.

Future research priorities include prospective, ideally randomized or well-matched comparative studies of ASA versus isolated Bankart repair and versus remplissage in patients with subcritical bone loss, preferably conducted by groups independent of the technique’s originators; longer-term follow-up (minimum 5–10 years) to assess durability and the development of osteoarthritis; standardized reporting of glenoid bone loss measurement methodology, thresholds, and recurrence definitions (dislocation, subluxation, and apprehension); prospective documentation of rehabilitation milestones, external-rotation loss, and return to sport; and dedicated, adequately powered studies evaluating ASA, with and without bone grafting, in patients with seizure disorders.

## 12. Conclusions

Arthroscopic subscapularis augmentation is a proposed adjunct to Bankart repair for selected patients with anterior shoulder instability and additional risk factors, including capsular deficiency, hyperlaxity, contact- or collision-sport participation, and glenoid bone loss within the ranges studied. Published observational cohorts report favorable short- to intermediate-term outcomes, but heterogeneous indications, limited comparative adjustment, inconsistent outcome definitions, and possible cohort overlap restrict conclusions about its incremental benefit relative to isolated Bankart repair, remplissage, or the Latarjet procedure. ASA provides an additional anterior soft-tissue restraint that is conceptually related to, but not established as mechanically equivalent to, the conjoint-tendon sling of the Latarjet procedure. Bone-graft-combined techniques, which are supported only by small series, should be considered separately, with graft resorption and other adverse outcomes fully characterized. Evidence for seizure-related instability remains limited to a single case report. Prospective comparative studies with standardized outcome definitions and longer follow-up are needed before treatment thresholds or claims of equivalent efficacy or superior safety can be established.

## Figures and Tables

**Table 1:** Indications and patient-selection criteria for arthroscopic subscapularis augmentation, by study.

Key Exclusions	Population	Tissue/Laxity Criterion	GBL Threshold	Study
GBL > 25%, MDI, osteoarthritis, overhead athletes	Contact/collision sport athletes, incl. revision cases	Capsulolabral insufficiency + Hill-Sachs lesion	<25%	Maiotti et al., 2016 [16]
GBL > 25%, MDI, osteoarthritis, overhead athletes	Contact sport athletes, min. 2-yr follow-up	Anterior capsular deficiency + Hill-Sachs lesion	<25% (range 5–23%; mean 12.8%)	Maiotti et al., 2017 [17]
GBL > 15%, MDI, osteoarthritis, throwing athletes	Collision/contact athletes	Documented hyperlaxity (Neer sulcus, Coudane-Walch > 85°)	<15% (mean 8.9%)	Maiotti et al., 2021 [18]
GBL > 15%, throwing sport, ISI < 3	Competitive/contact athletes	Instability Severity Index (ISI) score > 3	<15%	Maiotti et al., 2023 [21]
GBL > 25%, posterior/MDI instability, SLAP lesion, additional shoulder trauma, Hill-Sachs requiring surgery, glenohumeral osteoarthritis	Mixed, non-selected recurrent instability, incl. multi-dislocation history	Ligament-labral (capsulolabral) failure	<25% (mean 21%)	Karakus & Oztermeli, 2023 [22]
Previous shoulder surgery	Critical bone loss cohort	Hyperlaxity, >5 dislocations	15–30% (critical)	Russo et al., 2021 [23]
GBL < 10%, MDI, arthritis, <24-mo follow-up	Chronic instability, age ≥ 18 years	Anterior capsular insufficiency + engaging Hill-Sachs lesion	>10% (mean 18.5%)	Maiotti & Massoni, 2023 [20,24]
MDI, HAGL/Kim’s/ALPSA lesions, rotator cuff tear, no remplissage for engaging Hill-Sachs	Non-athletes, low-to-moderate activity, age < 60 years	None required beyond standard Bankart criteria	<20% (mean 6.5–7.6%)	Samant & Wade, 2023 [25]
GBL ≥ 13.5%	Recurrent anterior instability; center independent of the Maiotti–Russo group	NR	<13.5% (subcritical)	Brilakis et al., 2025 [26]

GBL, glenoid bone loss; MDI, multidirectional instability; ISI, Instability Severity Index. GBL thresholds are the eligibility criteria of each study, measured with different methods, and should not be interpreted as validated indications. NR, not reported. The single case report of ASA for seizure-related dislocation [27] is discussed separately in Section 10 and is not listed here as an established indication.

**Table 2:** Surgical technique variants of arthroscopic subscapularis augmentation and related biceps-based procedures.

Source	Graft/Fixation	Core Steps	Variant
**A. Native-tendon ASA (tenodesis of the upper subscapularis) and fixation variants**			
Maiotti & Massoni, 2013 [13]	Knotless suture anchor + tape/suture	Bankart repair + tenodesis of the upper one-third of the subscapularis tendon to the anterior glenoid neck.	Original ASA
Karakus & Oztermeli, 2023 [22]	Knotless suture anchors (2), suture lasso, sliding slipknot	The subscapularis tendon and the anteroinferior capsule are passed together in a single motion with a suture lasso through two knotless anchors (rather than augmenting the subscapularis as a separate second stage after capsule repair), fixed as one composite tissue layer.	Combined tendon-capsule single-pass ASA
Ren et al., 2023 [28]	Cortical button, adjustable-loop suture	Subscapularis upper-third tenodesis secured via a transglenoid tunnel using an adjustable-length loop cortical suspensory fixation device.	Transglenoid fixation ASA
**B. Graft-modified ASA**			
Ren et al., 2019 [29]	Tendon graft (autograft/allograft)	Subscapularis tenodesis using an interposed tendon graft combined with capsulolabral reconstruction.	Modified ASA (MASA)
**C. Bone-graft-combined ASA**			
Russo et al., 2018 [30]; Russo et al., 2021 [23]	Iliac crest autograft/allograft, cortical buttons	Bankart repair + glenoid bone graft fixed via transosseous tunnels/buttons, combined with upper-third subscapularis tenodesis at a higher anchor position.	ASA + arthroscopic bone graft
Maiotti & Massoni, 2023 [24]; Maiotti et al., 2023 [20]	Equine-derived xenograft, cerclage fixation	Glenoid bone block augmentation using a xenograft, secured with a posterior cerclage guide, combined with ASA.	ASA + xenograft glenoid bone block
**D. Biceps-based dynamic anterior stabilization (related to but distinct from ASA)**			
Huang et al., 2022 [31]	Biceps tendon autograft, tunnel fixation	The long head of the biceps tendon is tenotomized, braided, and passed through a scapular/subscapularis tunnel to re-create the sling effect.	LHBT-based “ASA” (ASA-LHBT)
Mehl et al., 2019 [15] (biomechanical validation)	Interference screw (inlay) or suture anchor/double-pulley (onlay)	The long head of biceps tendon is transferred through a subscapularis split to the anterior glenoid, with or without concomitant Bankart repair.	Long head of biceps-based Dynamic Anterior Stabilization (DAS)

ASA, arthroscopic subscapularis augmentation; DAS, dynamic anterior stabilization; LHBT, long head of the biceps tendon; MASA, modified arthroscopic subscapularis augmentation.

**Table 3:** Recurrence rates: isolated arthroscopic Bankart repair versus arthroscopic subscapularis augmentation.

Rowe (Pre → Post) or Other Outcome	Recurrence	Follow-Up	GBL	*n*	Study/Procedure
—	Reported up to 67% with substantial bone deficiency [1]; ISI > 6 associated with ~70% recurrence [2,3]	Variable	Variable, incl. bone loss	—	Isolated arthroscopic Bankart repair, high-risk cohorts
—	125/528 = 23.7%; GBL ≥ 16% predictive of recurrence	Variable	Mean 10.9%	528	Isolated arthroscopic Bankart repair, patients with GBL (contemporary pooled data) [4]
58.9 → 94.1	3/89 = 3.3%	31.5 mo (25–60)	<25%	89	Maiotti et al., 2016, ASA [16]
57.4 → 95.3	3/110 = 2.7%	40.5 mo (24–65)	<25% (mean 12.8%)	110	Maiotti et al., 2017, ASA [17]
68.5 → 92.7 ^a^; ASES 71.5 → 97.4	16/397 = 4.0% (9 traumatic, 7 atraumatic)	30.5 mo (24–109)	<15% (mean 8.9%)	397	Maiotti et al., 2021, ASA (hyperlaxity) [18]
89.2% excellent; WOSI 838 → 235	4/57 = 7.0%	24 mo–6 yr	<15%	57	Maiotti et al., 2023, ASA arm [21]
79.9% excellent; WOSI 1146 → 465	4/65 = 6.1%	24 mo–6 yr	<15%	65	Maiotti et al., 2023, remplissage arm [21]
33.3 → 82.6 (*p* = 0.001)	2/83 = 2.4%	32 mo (min 26)	<25% (mean 21%)	83	Karakus & Oztermeli, 2023 [22]
NR	2/34 = 5.9%	42 mo (mean)	<13.5%	34	Brilakis et al., 2025, ASA [26]
Greater ER deficit with ASA	0/17 = 0% (with ASA) vs. 2/39 = 5.1% (without ASA)	NR	<15%	17 vs. 39	Hantes et al., 2026, Bankart + remplissage ± ASA [33]
Less ER loss with LHBT transposition	0/25 vs. 0/25 at 1 yr	20 mo (13–28)	13.5–20%	25 vs. 25	Wang et al., 2025, Bankart + ASA vs. LHBT transposition [34]
NR	MASA group numerically superior to Bankart-repair-alone group	>24 mo	<25%	49 (46 f/u)	Ren et al., 2019, MASA vs. Bankart repair [29]
Postop median only: CB 75 (65–85) vs. SB 100 (100–100), *p* < 0.001	0/15 = 0% in both groups; recurrence not differentiated by technique in this non-athlete cohort	48 mo	<20% (mean 6.5% vs. 7.6%)	15 vs. 15	Samant & Wade, 2023, SB vs. CB [25]

Scores are means (preoperative → postoperative) unless otherwise stated; follow-up is the mean (range); recurrence is given as *n*/N = %. NR, not reported; —, not applicable. The first two rows are historical or pooled data for isolated Bankart repair and are not concurrent controls; direct comparisons between procedures are limited to [21,25,29,33,34]. ^a^ Value from Table 2 of the full text; the abstract of the same article reports 92.5. Recurrence definitions differed across series: redislocation (traumatic or atraumatic) in the Maiotti series [16,17,18] and in Brilakis et al. [26]; “recurrence” without further specification in the four-arm multicenter comparison [21]; redislocation or revision surgery in Samant and Wade [25]; and recurrent instability as reported by the authors in [22,29,33,34]. Subluxation and persistent apprehension were not consistently counted as failures. The Maiotti cohorts overlap in surgeons, centers, and study periods and should not be treated as independent samples. GBL, glenoid bone loss; ISI, Instability Severity Index; MASA, modified arthroscopic subscapularis augmentation; SB, subscapularis-augmented Bankart repair; CB, capsulolabral Bankart repair; WOSI, Western Ontario Shoulder Instability Index.

**Table 4:** Bone-graft-combined subscapularis augmentation compared with Latarjet-procedure outcome data.

Functional Outcome	Recurrence	Follow-Up	GBL	*n*	Study/Procedure
Rowe: 50 → 91.6 (ASA) vs. 50 → 89.8 (Latarjet); Constant: 55.2 → 93.8 vs. 56.2 → 93.3	0/20 (ASA; 1 transient subluxation) vs. 1/20 (Latarjet; coracoid nonunion)	20 mo (ASA); 21 mo (Latarjet)	Moderate (5–23%)	20 vs. 20	Russo et al., 2017, ASA vs. open Latarjet [19]
Constant: 82.9 → 88.9 (*p* = 0.002); Rowe: 25.3 → 89.1 (*p* < 0.001); UCLA: 23.7 → 31.5 (*p* < 0.001); VAS: 3.2 → 1.3 (*p* < 0.001); graft resorption in 100% (partial 9, complete 10)	2/19 = 11%	34.6 mo (24–48)	20 ± 3% (range 15–30%)	19	Russo et al., 2021, ASA + iliac crest allograft [23]
Rowe 38.3 → 95.5; WOSI 1242 → 120	0/20; no graft volume reduction on CT at 24 mo	24 mo (20–32)	Mean 18.5%	20	Maiotti et al., 2023, ASA + xenograft block [20]
98.5% excellent; WOSI 1456 → 319; highest complication rate (~15%)	1/66 = 1.5%	24 mo–6 yr	<15%	66	Maiotti et al., 2023, open Latarjet arm [21]
81.6% excellent; WOSI 1250 → 221	0/38 = 0%	24 mo–6 yr	<15%	38	Maiotti et al., 2023, Arthro-Latarjet arm [21]
Higher Rowe scores, less external-rotation loss than Bankart repair	11.6% (pooled Latarjet) vs. 21.1% (pooled Bankart repair)	Variable	Variable/severe	379 (pooled)	Latarjet, systematic review data [9]
NR	Open Latarjet: significantly lower redislocation than arthroscopic Bankart repair (*p* < 0.001), but more complications (*p* = 0.002)	Long-term	Variable	Pooled	Latarjet vs. arthroscopic Bankart, long-term review [10]

Scores are means (preoperative → postoperative) unless otherwise stated; “excellent” denotes the proportion of patients with an excellent Rowe score; follow-up is the mean (range). The last two rows are pooled systematic-review data for the Latarjet procedure and Bankart repair and are not direct comparisons with ASA; direct comparisons with ASA are limited to [19,21]. NR, not reported. ASA, arthroscopic subscapularis augmentation; CT, computed tomography; GBL, glenoid bone loss; UCLA, University of California, Los Angeles score; VAS, visual analog scale; WOSI, Western Ontario Shoulder Instability Index.

**Table 5:** Reported complication burden: bone-graft-combined ASA versus Latarjet-procedure literature.

Notes	Reported Complications	*n*	Study/Data Source
Graft resorption (100%) reported separately, not classified as a complication	0% perioperative complications; no graft fracture, malposition, neurovascular injury, or conversion to open surgery.	19	Russo et al., 2021, ASA + iliac crest allograft [23]
Highest complication rate among the 4 techniques compared in this multicenter study	Approximately 15%.	66	Maiotti et al., 2023, open Latarjet arm [21]
Soft-tissue arms; recurrence 7.0% and 6.1%, respectively	Lower than open Latarjet arm, as reported by the authors.	57 + 65	Maiotti et al., 2023, ASA + remplissage arms [21]
90% open technique; Level IV evidence pooled	30% overall complication rate; 7% unplanned reoperation; hardware complications 6.5% (open) to 17.6% (arthroscopic).	1904 (pooled, 45 studies)	Griesser et al., 2013, Bristow-Latarjet systematic review [35]
Illustrates that complication rates are highly surgeon/center-experience-dependent	4.3% overall 90-day complication rate (chiefly hematoma); 0% neurologic or hardware complications.	441	Scanlon et al., 2020, high-volume Latarjet center [36]

Complication rates are proportions of patients. ASA, arthroscopic subscapularis augmentation.

**Table 6:** Design and main characteristics of the clinical series included in this review.

Recurrence Definition	Patients Analysed; Follow-Up	Comparator	Recruitment Period; Centers	Design	Study
Post-traumatic redislocation	89 of 250 operated (161 excluded for insufficient follow-up); 31.5 mo	None	2010–2013; 4 centers	Retrospective case series	Maiotti et al., 2016 [16]
Traumatic redislocation	110; 40.5 mo	None	2010–2015; 4 centers	Retrospective multicenter case series	Maiotti et al., 2017 [17]
Redislocation, traumatic or atraumatic	397 of 591 screened; 30.5 mo	None	2009–2017; 6 centers	Retrospective multicenter case series	Maiotti et al., 2021 [18]
“Recurrence” (not further defined)	226; 24 mo–6 yr	Remplissage; open and arthroscopic Latarjet (non-randomized; baseline WOSI differed)	NR; multicenter	Retrospective multicenter comparison of 4 procedures	Maiotti et al., 2023 [21]
Dislocation; subluxation reported separately	20 vs. 20; 20–21 mo	Open Latarjet	2011–2015; single unit	Retrospective comparison of two series	Russo et al., 2017 [19]
Recurrent dislocation	19; 34.6 mo	None	NR	Case series	Russo et al., 2021 [23]
Recurrent dislocation	20; 24 mo	None	2019–2020; 2 hospitals	Case series	Maiotti et al., 2023 [20]
NR	83; 32 mo	None	NR; single surgeon	Single-surgeon series	Karakus & Oztermeli, 2023 [22]
NR	46 of 49; >24 mo	Bankart repair alone	NR	Retrospective comparison	Ren et al., 2019 [29]
Redislocation or revision surgery	15 vs. 15; 48 mo	Capsulolabral Bankart repair	NR	Comparison of two groups	Samant & Wade, 2023 [25]
Redislocation	34; 42 mo	None	NR; independent center	Case series	Brilakis et al., 2025 [26]
NR	17 vs. 39; NR	Bankart repair with remplissage without ASA	2018–2022	Retrospective cohort	Hantes et al., 2026 [33]
Recurrent dislocation	25 vs. 25; 20.1 mo	LHBT transposition with subscapularis augmentation	NR	Comparison of two groups	Wang et al., 2025 [34]
NR	36; short term	None	2022–2023; single hospital	Cross-sectional study	Hossain et al., 2025 [32]

NR, not reported. Information on consecutive recruitment and adjustment for baseline differences was rarely available in the published reports. ASA, arthroscopic subscapularis augmentation; GBL, glenoid bone loss; LHBT, long head of the biceps tendon; MASA, modified arthroscopic subscapularis augmentation. Follow-up is the mean unless otherwise stated.

## Data Availability

No new data were created or analyzed in this study. Data sharing is not applicable to this article.

## Abbreviations

The following abbreviations are used in this manuscript:

ASA	arthroscopic subscapularis augmentation
GBL	glenoid bone loss
ISI	Instability Severity Index
ASES	American Shoulder and Elbow Surgeons score
WOSI	Western Ontario Shoulder Instability Index
UCLA	University of California, Los Angeles shoulder score
VAS	visual analog scale
MASA	modified arthroscopic subscapularis augmentation
LHBT	long head of the biceps tendon
DAS	dynamic anterior stabilization
MDI	multidirectional instability
SB	subscapularis-augmented Bankart repair
CB	capsulolabral Bankart repair
CT	computed tomography
MRI	magnetic resonance imaging
